# Pharmacological challenge with a serotonin 1D agonist in alcohol dependence

**DOI:** 10.1186/1471-244X-5-31

**Published:** 2005-08-24

**Authors:** Bavanisha Vythilingum, Charmaine J Hugo, J Stefan Maritz, Willie Pienaar, Dan J Stein

**Affiliations:** 1 MRC Unit on Anxiety and Stress Disorders, Dept of Psychiatry University of Stellenbosch, South Africa; 2 Dept of Psychiatry, University of Stellenbosch, South Africa; 3 Biostatistics Unit, Medical Research Council of South Africa

## Abstract

**Background:**

Both animal and clinical studies have implicated serotonergic dysfunction in the pathogenesis of alcohol abuse and dependence. However the exact mechanisms involved remain unknown. Theoretically, low serotonin promotes alcohol seeking behavior. Sumatriptan is a serotonin1D agonist. It is postulated that sumatriptan's agonism at this terminal autoreceptor increases negative feedback, creating a net effect of decreased serotonergic neurotransmission. Administration of sumatriptan should therefore produce a craving for alcohol and the desire to drink.

**Methods:**

Fifteen patients with alcohol dependence who had undergone detoxification were recruited. Sumatriptan (100 mg) and placebo was administered in cross-over fashion on 2 separate days 72 hours apart. Both patients and raters were blind to all treatments.

Patients were assessed on the following scales at -30, 0, 30, 90, 150 and 210 minutes: A 6-item scale designed to rate the patient's intention to drink; The Sensation Scale; a 13-item affect analog scale designed to rate the pattern and extent of emotional changes; and an 8-item scale designed to rate the patient's craving for alcohol

**Results:**

No significant differences were found between the placebo and sumatriptan groups and no significant cross over effects were found.

**Conclusion:**

The general lack of efficacy of sumatriptan in producing alcohol-like symptoms or a desire to drink alcohol may suggest that the 5HT1D receptor plays little role in the pathophysiology of alcoholism.

## Background

Both animal and clinical studies have implicated serotonergic dysfunction in the pathogenesis of alcohol abuse and dependence [1,2]. However the exact mechanisms involved remain unknown. Pharmacological challenge studies provide one way of identifying the specific receptor systems involved.

Serotonergic dysfunction is thought particularly to be involved in alcohol craving. Verheul et al [3] proposed three types of craving – reward craving, relief craving and obsessive craving. Obsessive craving is defined as the loss of control over intrusive thoughts about alcohol and is believed to be mediated by a deficit of serotonin.

m-chlorophenylpiperazine (m-CPP) is a serotonin agonist with actions at 5-HT1a, 5-HT1d, 5-HT2c receptors. The 5HT1a and 5HT1d receptors are autoreceptors and agonism here has been demonstrated to decrease net serotonergic transmission.[4] m-CPP has been reported to elicit craving for alcohol and feelings similar to intoxication in abstinent alcoholics [5,6]). This relates well to animal studies, where TFMPP administration (a compound structurally similar to mCPP) has discriminant stimulus properties similar to low doses of alcohol [7].

Taken together, this suggests that alcohol may be used to self-medicate in patients with dysequilibrium of the serotonergic system. This hypothesis is given further support by studies showing the efficacy of serotonergic agents in the treatment of alcoholism [8-11]. The serotonin system is, however, complex, and mCPP is a relatively nonselective agent. In order to delineate further the exact mechanisms involved in alcoholism more selective agents must be used.

Sumatriptan is a selective serotonin(5HT)1D agonist. It is postulated that sumatriptan's agonism at this terminal autoreceptor increases negative feedback, creating a net effect of decreased serotonergic neurotransmission [4] In line with Verheul's model of craving [3] sumatriptan administration, by decreasing serotonergic transmission, should theoretically produce a desire to drink.

Studies of sumatriptan administration in alcohol dependent patients suggest involvement of the 5HT1D receptor. Sumatriptan stimulates the release of growth hormone (GH) and inhibits the release of prolactin in normal individuals. In both long term and newly abstinent alcoholics sumatriptan administration did not increase GH secretion, suggesting alteration at the 5HT1D receptor. [12,13]

The 5HT1D receptor may also be associated with repetitive behaviours. In autism, increased GH response to sumatriptan was noted [14], with the severity of repetitive behaviours correlating with response. Addictive behaviours may be viewed as a type of repetitive behaviour.

Furthermore, in OCD, sumatriptan has been suggested to produce similar effects to those of mCPP, and its administration led to exacerbation of symptoms [15], i.e loss of control over obsessive thoughts. We hypothesized that effects of sumatriptan would be similar to those of mCPP in patients with alcoholism, namely that administration of sumatriptan would potentiate obsessive craving for alcohol. Should this hypothesis be bourne out, it would point to specific involvement of the 5HT1d receptor in the pathophysiology of alcohol craving.

## Methods

Fifteen patients with alcohol dependence who had undergone detoxification (4 females and 11 males) were recruited from the acute detoxification units at Tygerberg and Stikland hospitals, Cape Town, South Africa. All subjects were medically stable, with no history of cardiac disorder, and with liver enzymes less than 1.5 times the maximum laboratory range at baseline. All subjects had at least seven alcohol and benzodiazepine-free days. For 72 hours prior to the challenge, subjects adhered to a tyramine-free diet.

Informed written consent was obtained from all subjects and the ethics committee of the University of Stellenbosch approved the study. The research was carried out in accordance with the Declaration of Helsinki of the World Medical Association. During the study subjects were inpatients at the Alcohol Rehabilitation Unit at Stikland Hospital, and received ongoing psychotherapy. On completion of a 6 week program, subjects were discharged to a weekly outpatient therapy group. These measures were felt to be sufficiently containing to prevent experimentally induced relapse.

Sumatriptan (100 mg) and placebo was administered orally in cross-over fashion on 2 separate days 72 hours apart. Sumatriptan was chosen as the challenge agent as it has the least side effects of the triptans. A drawback though, is that it has poor penetration of the blood brain barrier. However, previous work [12,13] has showed altered hormonal responses in alcohol dependent patients, suggesting that sufficient amounts do cross over. Furthermore, in studies of OCD and related disorders, sumatriptan is the most commonly used challenge agent. The 100 mg dose was chosen as in previous work on OCD and OCD related disorders, 100 mg had been shown to increase obsessions and repetitive behaviours [14,15] Both patients and raters were blind to all treatments.

Patients were assessed on the following scales at -30, 0 30, 90, 150 and 210 minutes:

1. A 6-item scale designed to rate the patient's intention to drink. (modified from [6]) Items were rated from 0 (not at all) to 4 (extremely), and included questions such as "I would like to drink alcohol" and "I intend to drink alcohol in the near future". A 6^th ^question, "the tablet I took this morning feels similar to alcohol" was also posed.

2. The Sensation Scale [16]. Items included side effects commonly produced by alcohol, such as nausea, facial flushing, dizziness and numbness. Items were rated from 0 (not at all) to 10 (a great deal).

3. A 13-item affect analog scale designed to rate the pattern and extent of emotional changes[17].

4. An 8-item scale designed to rate the patient's craving for alcohol. Items included " I crave a drink right now" and "I want a drink so bad I can taste it". [18]

Statistical analyses were carried on out the total score for each questionnaireas well specific variables of interest. For every variable the six sequential observed scores, y_1_...y_6_, of every subject on every occasion were summarized in two informative statistics, the sum S = y_1_+y_2_+y_3_+y_4_+y_5_+y_6_, and the linear trend contrast L = -8y_1_-5y_2_-2y_3_+y_4_+4y_5_+10y_6_. Note that the weights in the L contrast are obtained from the first order orthogonal polynomial for the unequally spaced time intervals of observation.

Thus, for a particular variable every subject has two values of S one for sumatriptan and one for placebo,, say S1 and S2., and the drug effect is measured by S1-S2. The cross-over effect is measured by an estimate of the difference in location of the distribution of S1-S2 in the two groups; it can be mean(S1-S2, Group 1)-mean((S1-S2, Group2), or an estimate based on the Wilcoxon Rank Sum test; there are other possibilities, not used here. In the case of the mean the natural test statistic is of the form z = (difference of means)/(s.e. of the difference); note that the Welch version of the denominator was used in the calculation of z. The distribution of this statistic is "t" if the variable S1-S2 is normally distributed, with appropriate adjustment of the number of degrees of freedom for the Welch version of the ttest. In the present instance it is not, but being a difference of linear functions the distribution is symmetric, and significance levels given by the t-distribution are robust. However, in marginal cases exact significance levels for z was obtained from a permutation distribution. Further, in marginal cases the Wilcoxon Rank Sum test was used for confirmation, or otherwise, of the result.

The linear effect scores, L, were analysed in the same way as the S scores.

## Results

All but 2 measures showed no significant changes (See table 1). On the variable "anxious" (item 8 of the affect analog scale) a significant cross over effect was found (z = -2.36, p = 0.046, df = 8; exact permutation P = 0.031; Wilcoxon Rank Sum test P = 0.023). In the group that received sumatriptan first the mean S1-S2 was negative, in the group that received placebo first it was positive. Neither mean differs clearly significantly from zero. In the placebo first group the signficance level attained was 0.08. (See table 2)

**Table 1:** 

		Group	N	Mean	SE Mean	T	p
Affect analog Scale	Cross-over effect	Placebo first	9	0.0	1.3	-.1.25	0.241
		Sumatriptan first	6	2.5	1.5		
	Linear Trend effect	Placebo first	9	-3.7	1.3	-0.91	0.391
		Sumatriptan first	6	-4.5	1.5		
Intent to drink scale	Cross Over effect	Placebo first	9	-2.3	4.5	-0.63	0.54
		Sumatriptan first	6	0.67	1.7		
	Linear Trend effect	Placebo first	9	16.1	7.9	2.12	0.058
		Sumatriptan first	6	-2.67	4.0		
Sensation Scale	Cross Over effect	Placebo first	9	-6.6	24	-0.66	0.52
		Sumatriptan first	6	11	11		
	Linear Trend effect	Placebo first	9	-6.8	21	0.80	0.44
		Sumatriptan first	6	-27.7	15		
Craving Scale	Cross Over effect	Placebo first	9	-7.4	4.2	-1.92	0.084
		Sumatriptan first	6	2	2.4		
	Linear Trend effect	Placebo first	9	3.6	5.9	-0.37	0.72
		Sumatriptan first	6	6.3	4.2		

**Table 2:** Cross Over Effect – variable "Anxious" on Affect Analog Scale

		Group	N	Mean	SE Mean	T	p
"Anxious"	Cross-over effect	Placebo first	9	-0.67	0.33	-2.36	0.046
		Sumatriptan first	6	0.83	0.54		

On the intention to drink scale no significant mean effect was found. In the linear trend scores there is some evidence of a significant crossover effect (t = 2.12, p = 0.058, df = 11; exact permutation P = 0.075; Wilcoxon Rank Sum test P = 0.059). Further analysis showed that no significant difference in linear trend was found for the group that received sumatriptan first, but in the group that received placebo first, a marginally significant linear trend effect was noted (t = 2.039, two-sided p = 0.07, one-sided p = 0.04) (See table 3)

**Table 3:** Linear Trend – Intent to Drink Scale

		Group	N	Mean	SE Mean	T	P
Intent to drink	Linear Trend effect	Placebo first	9	16.1	7.9	2.039	0.04
		Sumatriptan first	6	-2.67	4.0	-.066	0.53

## Discussion

Our results show that overall sumatriptan has no significant ability to provoke alcohol lsymptoms or to cause a desire or craving for alcohol. The significant placebo effect on the variable "anxious" may merely represent normal anxiety when confronted with an unfamiliar task. This is made especially plausible by the fact that it only occurs in the group that took placebo first, suggesting that as patients become more familiar with the trial the anxiety disappears.

The interpretation of the linear trend on the intention to drink scale in patients who received placebo first is more difficult. On its own this suggests that sumatriptan did cause changes not seen with placebo. However, if this was true, one would also expect to find a significant linear trend in the group who received sumatriptan first. Furthermore, a significant overall drug effect should have been found. It is more plausible that the trend found is due either to error introduced by the small group size and multiple tests, or by factors unrelated to sumatriptan within the group.

The general lack of efficacy of sumatriptan in producing alcohol-like symptoms or a desire to drink alcohol may suggest that the 5HT1D receptor plays little role in the pathophysiology of alcoholism. This is in contrast to studies that show decreased GH response to sumatriptan administration in abstinent alcoholics.

However, several limiting factors must be borne in mind. Firstly, the sample size was small and consisted of recently detoxified alcoholics. Chronic use of alcohol may alter the neurochemical milieu and provide a false picture of receptor involvement in alcoholism [1].

Secondly, sumatriptan has poor penetration of the blood-brain barrier, and its failure to provoke a response may be a result of this, rather than lack of involvement of the 5HT1D receptor in the pathogenesis of alcoholism. However, given that previous work has shown hormonal (in alcohol dependence) [12,13]and behavioural (in OCD) responses [15], this is less likely.

Furthermore, it may be that GH response to sumatriptan does not necessarily correlate with behavioural changes. In OCD, there have been conflicting reports about symptom exacerbation following sumatriptan challenges, yet a decreased GH response is observed. It is possible that GH release is mediated via different pathways to behavioural effects. This is suggested by the finding in autism, where increased GH release correlated with severity of repetitive behaviours. As both OCD and alcohol dependence can be conceptualized as repetitive behaviours, this contrasts with findings in these disorders.

Finally, the majority of the sample (11 patients) had late onset alcohol dependence (age of onset > 25) using the von Knorring criteria [19]. George et al [20] found ethanol -like responses to mCPP were only found in the early onset group. It is possible that we did not observe a response in our sample as most had late onset alcohol dependence.

## Conclusion

While our study suggests that the 5HT1D receptor does not play a significant role in the pathogenesis of alcoholism, further studies with sumatriptan and with agents that have better blood-brain barrier penetration are needed in both alcohol dependant and non-alcohol dependent samples to consolidate this finding. Stratification of further samples into early and late onset alcohol dependence may also be of use.

## Competing interests

The author(s) declare that they have no competing interests.

## Authors' contributions

BV participated in the statistical analysis and prepared the manuscript for publication. CJH participated in the study design and carried out the patient ratings. WP participated in the preparation of the manuscript. JSM performed the statistical analysis. DJS conceived of the study, participated in the study design and participated in the preparation of the manuscript.

## Pre-publication history

The pre-publication history for this paper can be accessed here:


